# Anti-malarial activity of a non-piperidine library of next-generation quinoline methanols

**DOI:** 10.1186/1475-2875-9-51

**Published:** 2010-02-11

**Authors:** Erin Milner, William McCalmont, Jayendra Bhonsle, Diana Caridha, Jose Cobar, Sean Gardner, Lucia Gerena, Duane Goodine, Charlotte Lanteri, Victor Melendez, Norma Roncal, Jason Sousa, Peter Wipf, Geoffrey Stuart Dow

**Affiliations:** 1 Division of Experimental Therapeutics, Walter Reed Army Institute of Research, Silver Spring, MD, USA; 2 Department of Chemistry, University of Pittsburgh, Pittsburgh, PA, USA

## Abstract

**Background:**

The clinical utility for mefloquine has been eroded due to its association with adverse neurological effects. Better-tolerated alternatives are required. The objective of the present study was the identification of lead compounds that are as effective as mefloquine, but exhibit physiochemical properties likely to render them less susceptible to passage across the blood-brain barrier.

**Methods:**

A library of drug-like non-piperidine analogs of mefloquine was synthesized. These compounds are diverse in structure and physiochemical properties. They were screened in appropriate in vitro assays and evaluated in terms of their potential as lead compounds. The correlation of specific structural attributes and physiochemical properties with activity was assessed.

**Results:**

The most potent analogs were low molecular weight unconjugated secondary amines with no heteroatoms in their side-chains. However, these compounds were more metabolically labile and permeable than mefloquine. In terms of physiochemical properties, lower polar surface area, lower molecular weight, more freely rotatable bonds and fewer H-bond acceptors were associated with greater potency. There was no such relationship between activity and LogP, LogD or the number of hydrogen bond donors (HBDs). The addition of an H-bond donor to the side-chain yielded a series of active diamines, which were as metabolically stable as mefloquine but showed reduced permeability.

**Conclusions:**

A drug-like library of non-piperidine analogs of mefloquine was synthesized. From amongst this library an active lead series of less permeable, but metabolically stable, diamines was identified.

## Background

Traditionally anti-malarial drugs have been used for prophylaxis and/or treatment. The target product profiles for these indications are very different and developing drugs simultaneously for both indications has become more difficult in recent times [1]. A new approach, intermittent preventive treatment (IPT) is the prevention of morbidity or mortality due to malaria through the intermittent administration of a single dose treatment of a drug at full therapeutic doses to asymptomatic, otherwise healthy infants (IPTi), pregnant women (IPTp) and travelers (IPTt) [1-3]. Drugs for these indications can theoretically be used for malaria prophylaxis as well. Drugs for IPTx indications and prophylaxis should ideally exhibit a long half-life, be very well-tolerated and safe in pregnancy. Mefloquine exhibits two of these characteristics, but will likely not find use as an IPT drug because of the adverse CNS events observed at the treatment level doses [4] that may be required for IPT. However, this would presumably not be an issue for next generation analogs of mefloquine without such a liability.

The minimum target product profile for such compounds would be similar clinical effectiveness to mefloquine, a long half-life, and fewer adverse neurological events. In the context of a drug discovery program this translates to useful potency against mefloquine-resistant strains of *Plasmodium falciparum*, adequate metabolic stability, long half-lives *in vivo*, and superior performance in an appropriate screen for neurological effects. Since mefloquine accumulates in the central nervous system and has multiple CNS targets (see discussion in our earlier papers [5,6]), it is logical to reduce partitioning of mefloquine into the CNS rather than focus on a specific neurological target. It is well known that lower permeability through the blood-brain barrier can be engineered into a chemical scaffold through manipulation of such properties as lipophilicity, hydrogen bonds, polar surface area, molecular weight, acidity and molecular flexibility [7-10]. Ideally, such a next generation quinoline methanol would be more potent against mefloquine-resistant strains of *P. falciparum in vitro *than mefloquine and lack cross-susceptibility to mefloquine. However, since mefloquine is used effectively in combination with other drugs [11], which would likely also be the case for a next generation quinoline methanol, these traits are not requirements for the target product profile.

Previously, it was reported that opening of the piperidine ring at the 4-position of the quinoline scaffold is associated with improved potency and selectivity relative to mefloquine [12]. However, the Walter Reed Army Institute of Research archive contains relatively few analogs of this type associated with the 2,8-trifluoromethyl quinoline core. This scaffold, therefore, represented a logical starting point from which to synthesize a new library of 4-position next generation quinoline methanols [13]. Structure-activity relationships amongst selected compounds from this library were recently reported [13]. The present study describes the most active compounds from this library together with the physiochemical properties that characterize them. The data suggest that diamine quinoline methanols exhibit the physiochemical properties required to achieve a balance between potency, reduced blood-brain barrier penetration, and metabolic stability.

## Methods

### Library synthesis and physiochemical properties

**Figure 1:**
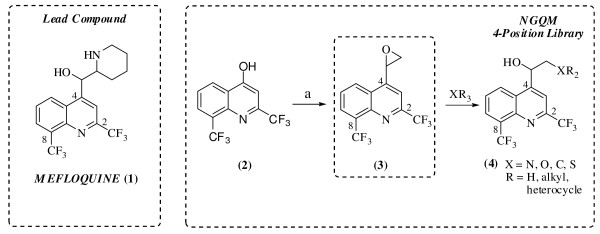
**Structure of mefloquine and synthesis of 4-position library**. The structure of mefloquine is indicated (1). The intermediate scaffold 4-(oxiran-2-yl)-2,8-bis(trifluoromethyl)quinoline (3) was synthesized from bis(trifluoromethyl)quinolin-4-ol (2) by the addition of POBr_3_, at 75°C - 150°C for 2 h with 91% yield. The resulting 4-bromo-2,8-bis(trifluoromethyl) quinoline was dissolved in tetrahydrofuran, cooled to -78°C and subjected to *n*-butyllithium. *N,N*-dimethylformamide was subsequently added to afford 2,8-bis(trifluoromethyl)quinoline-4-carbaldehyde. Utilization of Corey's dimethylsulfonium methylide provided racemic epoxide (3). The epoxide (3) can also be purchased commercially from Bioblocks (San Diego, California). The quinoline scaffold (3) was diversified at the 4-position in a single step with commercially available nucleophiles.

A library of one hundred ninety eight next generation quinoline methanols was synthesized. All analogs were modified at the 4-position. The synthesis was designed to provide rapid access to a broad range of chemotypes at the 4-position in a single step from the 4-(oxiran-2-yl)-2,8-bis(trifluoromethyl)quinoline scaffold using the general reaction scheme outlined in Figure 1.

Compounds were designed to be rule of 5 (RO5) compliant [14] and to encompass the widest feasible range of molecular weight (MW), LogD, LogP, freely rotatable bonds (FRBs), polar surface area (PSA), hydrogen bond donors (HBDs) and hydrogen bond acceptors (HBAs, see Table 1). All physiochemical properties were calculated using ACD (Version 10, ACD Labs, Toronto, Canada) except LogD (pH 7.4) which was determined using Pipeline Pilot. (Version 6.1, Accelrys, San Diego, California). These properties have been defined in detail elsewhere [15]. They numerically represent the size (MW), lipophilicity (LogD and LogP), molecular flexibility (FRBs) and H-bonding capacity (HBDs and HBAs) of a compound, all of which impact biological properties important in drug development.

**Table 1:** Physiochemical properties of the 4-position library

Parameter*	Range
MW	335-552
LogD	-1.2-6.9
PSA	36-111
LogP	-2.2-7.1
FRBs	3-12
HBDs	1-5
HBAs	3-8
LVs	0-1

* MW is molecular weight, PSA = polar surface area, FRBs = freely rotatable bonds, HBDs = H-bond donors, HBAs = H bond acceptors and LVs = Lipinski violations.

A R05 compliant compound was looked for, since molecules of this type are more likely to be orally bioavailable [14]. An R05 compliant compound is one that possesses at least three of the following properties; molecular weight < 500, LogP is < 5, HBDs < 5 and HBAs < 10. These breakpoints make sense given that good oral bioavailability is a function of solubility and permeability. Larger compounds (high MW) tend to be less permeable and less soluble. Greater lipophilicity (LogP) may improve permeability, however too much lipophilicity may reduce aqueous solubility. Compounds with more H-bonds (higher HBD and HBA counts) tend to have greater solubility, but this may come at the expense of lower permeability.

Limiting the physiochemical properties of a molecule to R05 space may have unintended effects on other parameters of interest. For example, a quinoline methanol could theoretically require a number of H-bonds in excess of R05 constraints if blood-brain barrier permeability is to be limited, but might be poorly bioavailable and lack anti-malarial activity if permeability across the intestine wall and parasite membranes is similarly curtailed. There may also be unintended pharmacokinetic consequences if greater polarity was associated with greater renal clearance. Therefore, the construction of a library with the maximum diversity of physiochemical properties should maximize the likelihood of identifying a lead series with the appropriate balance of biological properties.

### *Plasmodium falciparum *susceptibility assays

The *in vitro *activities of quinoline methanols against *P. falciparum *strains W2, D6, TM91C235, and TM90C2A were evaluated using the traditional labeled hypoxanthine assay of Desjardins *et al *[16] as modified by Milhous *et al *[17]. These four *P. falciparum *strains were selected since they have various levels of resistance to conventional anti-malarials. W2 is chloroquine resistant and mefloquine sensitive, D6 is chloroquine sensitive but naturally less susceptible to mefloquine, TM91C235 is resistant to mefloquine, chloroquine, and pyrimethamine as is TM90C2A, however this latter parasite is a two *pfmdr1 *copy strain (pfmdr1 amplification has been associated with clinical mefloquine resistance). Mefloquine is routinely screened in these assays to ensure the validity. Historical values for mefloquine against all the strains are reported in Table 1.

### Toxicity assays

General cytotoxicity was assessed by first determining LC_50 _'s against a rodent macrophage (RAW) cell line using the 3-(4,5-dimethylthiazole-2-yl)-2,5-diphenyltetrazolium bromide (MTT) assay as described in one of our earlier studies [5]. This screen was conducted to identify compounds that may require de-selection due to generalized toxicity. Mefloquine is routinely screened in this assay to ensure validity. Historical data for mefloquine is reported in Table 2. While the central nervous system targets of mefloquine are not known, direct neurocytotoxicity at high concentrations, as well as modulation of adenosine receptors, may play a role [18,19]. Selection of compounds with more favorable profiles than mefloquine against these targets may be advantageous, all other factors being equal. *In vitro *neurocytotoxicity was assessed by determining LC50s against primary mouse neurons using the MTT assay as described previously [5]. Inhibition of the A2A and A1 receptors was determined at 200 nM in duplicate by Caliper Biosciences (Hanover, Maryland).

**Table 2:** Anti-malarial and toxicity data for compounds selected for secondary *in vitro *screening.*

WR#	IC90 Pf (ng/ml)*				LC50 RAW (μM)**	LC50 neurons (μM)	% A2A inhibition at 200 nM	% A1 inhibition at 200 nM
				
	W2	D6	C235	C2A				
MQ	6.2 +/- 2.8 (532)	17 +/- 11(536)	52 +/- 30 (367)	74 +/- 32 (77)	9.0 +/- 3.7 (27)	41	62	4.0
308245	17	45	58	80	40	> 120	0	0
308255	3.8	15	19	37	58	> 120	NT	NT
308257	4.7	23	23	39	14	> 120	17	79
308266	6.1	25	24	52	13	59	NT	NT
308278	9.7	36	39	54	21	> 120	1	9.4
308396	6.2	29	40	48	18	52	0	2.9
308446	< 1	14	19	16	8.9	48	NT	NT
398387	1.2	7	12	11	21	> 120	NT	NT
308388	1.1	6.7	11	10	16	56	NT	NT
308413	1.1	12	15	7.5	8.5	> 120	NT	NT

* For mefloquine IC90s are presented as mean +/- standard deviation (n). ** RAW refers to the murine RAW macrophage cell line. For mefloquine LC50 is presented as mean +/- standard deviation (n).

### Permeability assay

Since mefloquine has multiple potential targets and the clinically relevant ones are not known, reduced blood-brain barrier penetration is a logical approach to reduce exposure of multiple targets to a next generation quinoline methanol. For this reason, the apparent permeability of selected compounds across MDR1-transfected MDCK cell monolayers [20] was determined. This assay is an *in vitro *surrogate of the blood-brain barrier.

Permeability was determined by Absorption Systems (Exton, PA). MDR1-MDCK cell monolayers were grown to confluence on collagen-coated, microporous, polycarbonate membranes in 12-well Costar Transwell plates. Data are considered valid for a specific assay plate if TEER values are < 1400 Ω cm^2^, the P_app _of propanolol is between 10-30 × 10^-6 ^cm/s and the P_app _of atenolol is < 0.5 × 10^-6 ^cm/s. The permeability assay buffer was Hanks Balanced Salt Solution containing 10 mM HEPES and 15 mM glucose at a pH of 7.4. A known p-glycoprotein inhibitor cyclosporin A (CSA) was also added to the assay buffer at 10 μM. Bovine serum albumin (1%) was added to the receiver well. The dosing solution concentrations of the test compounds were 5.0 μM in the assay buffer. All cell monolayers were first pre-incubated for 30 minutes with assay buffer. After 30 minutes, the buffer was removed, replaced with fresh buffer, and time was recorded as 0. The addition of BSA, pre-incubation, and use of a longer incubation time were employed to mitigate potential low recovery or permeability that is sometimes observed for lipophilic or 'sticky' compounds. Cell monolayers were dosed on the apical side (A-to-B) or basolateral side (B-to-A) and incubated at 37°C with 5% CO2 in a humidified incubator. After 2 hours, aliquots were taken from the receiver chambers. Samples were taken from the donor chamber at 0 and 2 hours. Each determination was performed in duplicate. The lucifer yellow flux was also measured for each monolayer to ensure no damage was inflicted to the cell monolayers during the flux period. All samples were assayed by LC/MS/MS using electrospray ionization.

Apparent permeability in the apical (A-B direction), Papp_A-B_, and percent recovery are reported. Apparent permeability is a measure of the rate of transport across the cell monolayer. Percent recovery refers to the amount of compound recoverable at the end of the assay. Low recovery may indicate non-specific binding to assay plates, instability or accumulation in the cell pellet. In the case of mefloquine, relatively low recovery is likely a consequence of accumulation in cell membranes [21,22] rather than non-specific binding.

The apparent permeability, Papp, and percent recovery were calculated as follows:

Papp = (*d*Cr/*d*t) × Vr/(A × C0) (1)

Percent Recovery = 100 × ((Vr × Cr^final^) + (Vd × Cd^final^))/(Vd × CN) (2)

where,

*d*Cr/*d*t is the slope of the cumulative concentration in the receiver compartment versus time in μM s^-1^.

Vr is the volume of the receiver compartment in cm^3^.

Vd is the volume of the donor compartment in cm^3^.

A is the area of the cell monolayer (1.13 cm^2 ^for 12-well Transwell).

C0 is the measured concentration of the donor chamber at time 0 in μM.

CN is the nominal concentration of the dosing solution in μM.

Cr^final ^is the cumulative receiver concentration in μM at the end of the incubation period.

Cd^final ^is the concentration of the donor in μM at the end of the incubation period.

### Metabolic stability and drug-drug interaction assays

Interesting compounds were also submitted for drug-drug interaction and metabolic stability assays. Low IC50s against specific cytochrome P450 isoforms may indicate the potential for a harmful drug interaction to occur *in vivo *with substrates of that isoform. A lack of metabolic stability *in vitro *may indicate potential metabolic instability *in vivo*, and, therefore, a shorter half-life. Drug interaction potential was evaluated *in vitro *using BD Gentest (Franklin Lakes, NJ) CYP450 inhibition kits as recommended by the manufacturer. Metabolic stability assessments were conducted using *in vitro *liver microsomes. Compound stocks at 10 or 20 mM (depending on solubility) in DMSO are diluted to a final concentration of 1 μM into a mixture containing, 0.5 mg/mL of pre-warmed pooled human or mouse liver microsomes (BD Gentest), 1.3 mM NADP (Sigma), 3.3 mM MgCl_2 _(Sigma), and 0.1 M pH 7.4 PBS using a TECAN Genesis robotic liquid handler. The reaction is started with the addition of 1 U/mL glucose-6-phosphate dehydrogenase G6PD. The mixture is incubated on a shaking platform at 37°C, and aliquots are taken and quenched with the addition of an equal volume of cold acetonitrile at 0, 10, 20, 30, and 60 min. Samples are centrifuged at 3700 rpm for 10dd min at 20°C to remove debris. Sample quantification is carried out by LC/MS, and metabolic half-life is calculated by log plots of the total ion chromatograph area remaining.

## Results

### Synthesis and primary screening

**Figure 2:**
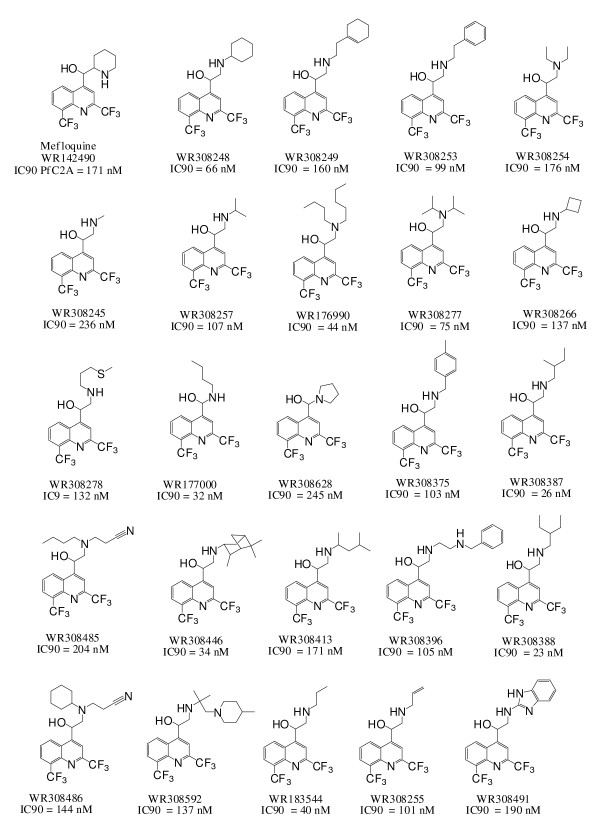
**Structures and IC90s of the most potent quinoline methanols**. IC90s are against the mefloquine resistant Pf C2A strain.

One hundred ninety eight quinoline methanols were synthesized. The ranges of physiochemical properties represented by the library are outlined in Table 1. The primary screening data and physiochemical properties of all compounds are presented in Additional File 1. Of the original 198 compounds, 24 (12%) exhibited IC90s less than 250 nM against mefloquine-resistant PfC2A and cytotoxicity similar or better than mefloquine. The structures of these compounds and their IC90s are presented in Figure 2. Ten of these compounds were selected for screening in various secondary assays based on their chemical structures. WR308278 and WR308396 were selected as they were the only compounds containing heteroatoms in the side-chain that were more potent than mefloquine. WR308245, WR308255, WR308257 and WR308266 were selected because they were approximately equivalent or superior to mefloquine in terms of potency, but contained less steric bulk in their side-chains. WR308387, WR308388, WR308413 and WR308446 were selected as they were substantially more potent than mefloquine *in vitro*.

### Secondary screening

The rank-order of mefloquine and quinoline methanols in terms of potency against the four strains of Pf was similar in most cases (Table 2). Most of the compounds screened were more metabolically labile than mefloquine (Table 3). The exceptions were those in which the side-chain was presumably less susceptible to N-dealkylation (WR308245 and WR308257) and the diamine WR308396. All compounds except WR308245 and WR308255 exhibited more potent inhibitory effects on cP450 2D6 than the other isoforms (Table 3). All the compounds were less neurotoxic than mefloquine. WR308245, WR308257, WR308278 and WR308387 were more permeable across MDCK-MDR1 cell monolayers than mefloquine (Tables 2 and 3). WR308396 exhibited slightly lower permeability than mefloquine and half the permeability of WR308387 (Table 3). The inhibition of the A2A and A1 receptors by four of the analogs at 200 nM was evaluated (Table 2). In most cases the level of inhibition observed was lower or comparable to that observed with mefloquine. The exception was WR308245 against the A1 receptor (Table 2).

**Table 3:** Metabolic stability, drug-drug interaction and permeability screening results for mefloquine and interesting next generation quinoline methanols

WR#	Half-life in mouse micro (min)	IC50 1A2 (uM)	IC50 2C9 (uM)	IC50 2C19 (uM)	IC50 2D6 (uM)	IC50 3A4 (uM)	**Papp**_A-B _MDCK-MDR1 in cells(% recovery)*
MQ	> 60	> 40	14	18	5.1	19	9.4 (< 40)**
308245	> 60	1.8	> 40	33	7.4	26	24 (59)
308255	25	1.5	25	25	2.9	18	NT
308257	> 60	3.9	37	26	1.9	> 40	25 (69)
308266	22	2.4	12	7.6	1.4	5.7	NT
308278	10	3.5	6.3	3.6	1.4	16	38 (54)
308396	> 60	5.4	14	5.8	0.7	3.3	8.4 (18)
308446	13	> 40	16	14	9.1	> 40	NT
398387	20	2.1	11	7	2	> 40	18 (42)
308388	16	1.2	13	3.4	0.8	33	NT
308413	19	26	19	5.8	1	> 40	NT

* Apparent permeability (Papp_A-B_) across MDR1-transfected MDCK cell monolayers in the apical (A-B) direction. Units are 10^-6 ^cm/s. % recovery is presented in brackets. ** Value for mefloquine is an average of n = 3 assays.

**Figure 3:**
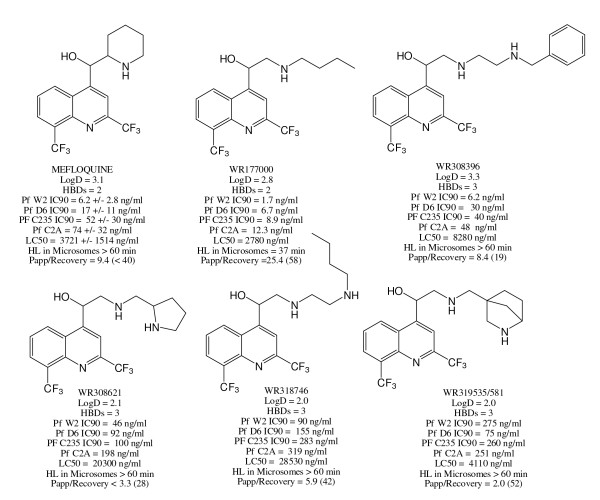
**Structures and key biological data for diamine quinoline methanols**. The *P. falciparum *IC90 and RAW macrophage LC50 data for mefloquine are presented as the mean +/- standard deviation. The LC50 values are against the macrophage cell line. Papp/recovery refers to the apparent permeability in the apical direction across MDR1-transfected MDCK cell monolayers (units in 10^-6 ^cm/s) and the % recovery for each compound at the end of the experiment.

On the basis of its equivalent potency and metabolic stability to mefloquine, and lower permeability, toxicity and inhibition A2A and A1 receptors, WR308396 appeared to be the most promising compound. Accordingly, a number of analogs of this lead structure were synthesized. These compounds were all active and exhibited improved metabolic stability and permeability relative to mefloquine (Figure 3). In some instances, cross-susceptibility profiles were different from mefloquine.

### Relationship between activity and physiochemical properties

Physiochemical properties amongst inactive compounds, active compounds with IC90s < 540 ng/ml or 1000 nM and active compounds with IC90s < 250 nM were compared. The threshold for inactivity was set at an IC90 > 540 ng/ml (or the approximate corresponding concentration of 1000 nM) since this was the highest concentration tested in the assay. There were no significant differences amongst these groups in terms of LogD, HBDs and LogP (Table 4). The most potent compounds exhibited significantly lower molecular weights, lower PSA and fewer HBAs compared to inactive compounds and fewer HBAs and lower molecular weights compared to compounds with active compounds. Active compounds exhibited significantly increased FRBs relative to inactive compounds (Table 4).

**Table 4:** Physiochemical properties of potent, active and inactive compounds

Parameter*	Active Compounds with IC90s < 250 nM	Active Compounds with IC90s < 540 ng/ml or 1000 nM	Inactive Compounds (IC90s > 540 ng/ml or 1000 nM)
	Mean +/- STDEV (N)	Mean +/- STDEV (N)	Mean +/- STDEV (N)
MW	410 +/- 35 (25)**#	444 +/- 37 (66)	435 +/- 45 (107)
LogD	3.6 +/- 0.77 (24)	3.6 +/- 1.2 (66)	3.9 +/- 1.3 (106)
PSA	48 +/- 9.6 (25)**	57 +/- 18 (66)**	66 +/- 19 (106)
LogP	3.5 +/- 0.88 (25)	3.4 +/- 1.2 (66)	2.9 +/- 1.5 (106)
FRBs	6.3 +/- 1.7 (25)	6.5 +/- 1.7 (66)**	5.8 +/- 1.8 (106)
HBDs	1.8 +/- 0.55 (25)	1.8 +/- 0.8 (66)	2.0 +/- 0.98 (106)
HBAs	3.2 +/- 0.52 (25)**#	4.0 +/- 0.89 (66)	4.4 +/- 1.3 (106)

* MW is molecular weight, PSA = polar surface area, FRBs = freely rotatable bonds, HBDs = H-bond donors, HBAs = H bond acceptors and LVs = Lipinski violations. Significant differences from inactive compounds or compounds with IC90s < 540 ng/ml/1000 nM# (one way ANOVA followed Bonferroni post-test, *P *< 0.05) are denoted by the symbols ** and # respectively.

### Structural characteristics of active and inactive compounds

Analogs were categorized based upon their structural motifs. The proportion of active (IC90 < 500 ng/ml or 1000 nM) and inactive compounds containing these functional groups was determined and differences between the groups were tested for significance using Fisher's Exact test (Table 5). The inactive group contained a greater proportion of compounds in which the 4-position amino side-chain contained additional heteroatoms, analogs in which the hydroxyl group or amine functionality were replaced, and compounds in which the first nitrogen atom in the side-chain was conjugated. The active groups of compounds contained a higher proportion of secondary amines and compounds in which the amino side-chain contained no additional heteroatoms. The inactive and active groups contained similar proportions of tertiary and benzyl amines.

**Table 5:** Proportions of different functional groups present in active and inactive amines

Chemical Property	Number of Analogs in Subset of Active or Inactive Amines*		P value**
		
	Active (# of 69) (%)	Inactive (# of 129) (%)	
N or OH Replacement	1 (1.4)	12 (9.3)	0.036
Conjugated Amines	9 (13)	49 (38)	0.0003
Benzyl Amines	15 (22)	19 (15)	0.238
Side-chain Contains Heteroatoms	36 (52)	96 (74)	0.0025
Tertiary Amines	20 (29)	31 (24)	0.4964
Secondary Amines	49 (71)	78 (60)	0.0129
All Carbon Side-chain	21 (30)	8 (6.2)	< 0.0001

* An active compounds is one with an IC90 < 1000 nM against *Pf*C2A. ** Fisher's exact, *P *< 0.05 considered significant.

## Discussion

In a previous study it was shown that non-piperidine analogs of mefloquine such as WR177000 were more potent and exhibited improved selectivity indices relative to mefloquine. In this study, it was observed that WR177000 and related compounds such as WR308336 and WR308387 share a set of structural features essential for good potency; these are low molecular weight, and unconjugated straight or branched-chain amines in which the 4-position hydroxyl group is retained. The library was structurally diverse within the constraints of drug-like space. This suggests it may not be feasible to synthesize quinoline methanols more potent than these whilst retaining the physiochemical properties associated with acceptable oral bioavailability.

As outlined previously and confirmed here, most quinoline methanols such as WR177000 and its analogs are metabolically labile. They also exhibit greater permeability than mefloquine across MDCK cell monolayers. In general, metabolic lability can be resolved by lowering lipophilicity [15]. Permeability across the blood-brain barrier can be reduced by increasing MW, HBDs, HBAs, PSA or lowering LogP or LogD. *A priori*, one might hypothesize that activity would be negatively affected by the same trends, since impermeable compounds are also likely to be inactive. Based on a global analysis of active and inactive compounds, only LogP, LogD and HBDs were not statistically associated with activity in the manner one might suspect. These observations lead to the hypothesis that within the constraints of drug-like space, potency is more easily retainable when the scaffold is modified to reduced blood-brain barrier permeability through H-bond donor addition (as in WR308396), rather than using the other strategies outlined above.

If so, diamine analogs of WR308396 would be expected to be active, metabolically stable and exhibit reduced permeability across MDCK cell monolayers. In fact, they exhibited similar metabolic stability to mefloquine and lower permeability relative to mefloquine and WR177000 analogs. All the diamines were active, albeit with somewhat reduced potency relative to mefloquine. Intriguingly, some of the cyclic diamines exhibited altered cross-susceptibility profiles relative to mefloquine. Based on these observations, diamine analogs of WR308396 appear to exhibit the best balance of desired biological properties within the constraints of drug-like space. A more detailed diamine SAR is currently being developed through additional analog synthesis and characterization in *in vivo *efficacy models and pharmacokinetic studies.

## Conclusion

Mefloquine is one of the few available drugs that could theoretically be used for malaria prophylaxis and IPT. However, this potential may not be realized due to its association with adverse CNS events at therapeutic doses. An analog that did not cross the blood-brain barrier would, therefore, have great utility. In this study, a library of drug-like, structurally diverse, non-piperidine analogs of mefloquine was synthesized. From this library an active series of diamines was identified with similar metabolic stability and lower permeability than mefloquine. These compounds have one additional H-bond donor compared to mefloquine. The *in vivo *efficacy and pharmacokinetics of these compounds are currently being investigated.

## Competing interests

The authors declare that they have no competing interests.

## Authors' contributions

All authors made intellectual contributions to this study through past or current membership of the Next Generation Quinoline Methanol project team. The objective of this project team is to identify a development candidate from the quinoline methanol class for malaria prophylaxis and IPT. EEM, WFM and GSD conceived the general project strategy and prepared the manuscript. EEM synthesized most of the analogs described in this study. SRG, WFM, PW, DG, JC synthesized or contributed to the synthesis of some of the analogs described in this study. DC executed the cytotoxicity and neurotoxicity assays. NR and LG executed the Pf susceptibility assays. JS and VM executed the metabolic stability and DDI assays. JB provided chemi-informatics and identified starting materials.

All authors read and approved the final manuscript.

## Supplementary Material

Additional file 1 **Primary screening data and physiochemical properties of next generation quinoline methanols**. This EXCEL file contains the structure number for 198 next generation quinoline methanols together with their calculated physiochemical properties, IC90 values against four strains of *P. falciparum*, and LC50 values against RAW macrophages. The compounds are broken out into three categories as follows; potent compounds (IC90 < 250 nM), active compounds (IC90s greater than 250 nM but less than either 1000 nM or 540 ng/ml) and inactive compounds (IC90s > 1000 nM or 540 ng/ml). For each of these three groups of compounds, the group means for each physiochemical parameter are presented together with standard deviations and sample size sizes. These data were used to perform the analyses outlined in Table 4. Click here for file

## References

[B1] Dow GS Magill AJ Ohrt C Clinical development of new prophylactic antimalarial drugs after the 5th Amendment to the Declaration of Helsinki Ther Clin Risk Manag 2008 4 19209263 10.2147/tcrm.s1025 PMC2621393

[B2] Schellenberg D Cisse B Mendendez C The IPTi Consortium: research for policy and action Trends Parasitol 2006 22 296 300 10.1016/j.pt.2006.05.006 16713739

[B3] Shanks GD Magill AJ Freedman DO Keystone JS Bradley DJ Steffen R Drug-free holidays: pre-travel versus during travel malaria chemoprophylaxis Am J Trop Med Hyg 2007 77 1 2 17620622

[B4] Rendi-Wagner P Noedl H Wernsdorfer WH Weidermann G Mikolasek A Kollaristch H Unexpected frequency, duration and spectrum of adverse events after therapeutic dose of mefloquine in healthy adults Acta Trop 2002 81 167 173 10.1016/S0001-706X(01)00210-8 11801224

[B5] Caridha A Yourick D Cabezas M Wolf L Hudson TH Dow GS Mefloquine-induced disruption of calcium homeostasis in mammalian cells is similar to that induced by ionomycin Antimicrob Agent Chemother 2008 52 684 693 10.1128/AAC.00874-07 PMC2224738 17999964

[B6] Dow G Bauman R Caridha D Cabezas M Du F Gomez-Lobo R Park M Smith K Cannard K Mefloquine induces dose-related neurological effects in a rat model Antimicrob Agent Chemother 2006 50 1045 1053 10.1128/AAC.50.3.1045-1053.2006 PMC1426433 16495267

[B7] CLARK DE In silico prediction of blood-brain barrier permeation Drug Discov Today 2003 8 927 933 10.1016/S1359-6446(03)02827-7 14554156

[B8] Doan KM Humphreys JE Webster LO Wring SA Shampine LL Serabit-Singh CJ Adkinson KK Polli JW Passive permeability and p-glycoprotein-mediated efflux differentiate central nervous system (CNS) and non-CNS marketed drugs J Pharmacol Exp Therap 2002 303 1029 1037 10.1124/jpet.102.039255 12438524

[B9] Pardridge WM Transport of small molecules through blood-brain barrier: biology and methodology Adv Drug Deliv Rev 1995 15 5 36 10.1016/0169-409X(95)00003-P 35524389

[B10] Pardridge WM CNS drug design based on principles of blood-brain barrier transport J Neurochem 1998 70 1781 1792 9572261 10.1046/j.1471-4159.1998.70051781.x

[B11] Price RN Uhleman AC Brockman A McGready R Ashley E Phaipun L Patel R Laing K Looareesuwan S White NJ Nosten F Krishna S Mefloquine resistance in *Plasmodium falciparum *and increased pfmdr1 gene copy number Lancet 2004 364 438 447 10.1016/S0140-6736(04)16767-6 15288742 PMC4337987

[B12] Dow GS Heady TN Bhattarchargee AK Caridha D Gerena L Gettayacamin M Lanteri CA Obaldia N III Roncal N Shearer T Smith PL Tungtaeng A Wolf L Cabezas M Yourick D Smith KS Utility of alkylaminoquinolinyl methanols as new antimalarial drugs Antimicrob Agents Chemother 2006 50 4132 4143 10.1128/AAC.00631-06 16966402 PMC1694001

[B13] Milner E McCalmont W Bhonsle J Caridha C Carroll D Gardner S Gerena L Gettayacamin M Lanteri C Luong T Melendez V Moon J Roncal N Sousa J Tangtaeng A Wipf P Dow GS Structure-activity relationships amongst 4-position quinoline methanol antimalarials that inhibit the growth of drug sensitive and resistant strains of *Plasmodium falciparum* Bioorg Med Chem Lett 2010 20097070 10.1016/j.bmcl.2010.01.001

[B14] Lipinski CA Drug-like properties and the causes of poor solubility and poor permeability J Pharmacol Toxicol Methods 2000 44 235 249 10.1016/S1056-8719(00)00107-6 11274893

[B15] Kerns EH Di L Drug-like properties: concepts, structure design and methods 2008 Burlington, MA, Elsevier Inc Dow GS, Chen Y, Andrews KT, Caridha D, Gerena L, Gettayacamin M, Johnson J, Li Q, Melendez V, Obaldia N III, Tran TN and Kozikowski A:**Antimalarial activity of phenylthiazolyl-bearing hydroxamate-based histone deacetylase inhibitors**. *Antimicrob Agents Chemother *2008 **52:**3467-3477.

[B16] Desjardins RE Canfield CJ Haynes JD Chulay JD Quantitative assessment of antimalarial activity in vitro by a semiautomated microdilution technique Antimicrob Agents Chemother 1979 16 710 8 394674 10.1128/aac.16.6.710 PMC352941

[B17] Milhous WK Weatherly NF Bowdre JH Desjardins RE In vitro activities of and mechanisms of resistance to antifol antimalarial drugs Antimicrob Agents Chemother 1985 27 525 30 3890727 10.1128/aac.27.4.525 PMC180089

[B18] Gillespie JJ Adams DR Bebbington D Benwell K Cliffe IA Dawson CF Dourish CT Fletcher A Gaur S Giles PR Jordan AM Knight AR Knutsen LJ Lawrence A Lerpiniere J Misra A Porter RH Pratt RM Shepherd R Upton R Ward S Weiss SM Williamson DS Antagonists of the human adenosine A2A receptor. Part 1: Discovery and synthesis of thieno[3,2-d]pyrimidine-4-methanone derivatives Bioorg Med Chem Lett 2008 18 2916 2919 10.1016/j.bmcl.2008.03.075 18406614

[B19] Weiss SM Benwell K Cliffe IA Gillespie RJ Knight AR Lerpiniere J Misar A Pratt RM Revell D Upton R Dourish CT Discovery of nonxanthine adenosine A2A receptor antagonists for the treatment of Parkinson's disease Neurology 2003 61 S101 106 14663021 10.1212/01.wnl.0000095581.20961.7d

[B20] Wang Q Rager JD Weinstein K Kardos PS Dobson GL Li J Hidalgo IJ Evaluation of the MDR-MDCK cell line as a permeability screen for the blood-brain barrier Int J Pharm 2005 288 349 359 10.1016/j.ijpharm.2004.10.007 15620875

[B21] Go ML Ngiam TL Thermodynamics of partitioning of the antimalarial drug mefloquine in phospholipid bilayers and bulk solvents Chem Pharm Bull 1997 45 2055 2060 9433777 10.1248/cpb.45.2055

[B22] Zidovetzki R Sherman IW Attiya A De Boeck H A nuclear magnetic resonance study of the interactions of the antimalarials chloroquine, quinacrine, quinine and mefloquine with dipalmitoylphosphatidylcholine bilayers Mol Biochem Parasitol 1989 35 199 207 10.1016/0166-6851(89)90206-5 2787476

